# Modulation of Calmodulin Lobes by Different Targets: An Allosteric Model with Hemiconcerted Conformational Transitions

**DOI:** 10.1371/journal.pcbi.1004063

**Published:** 2015-01-22

**Authors:** Massimo Lai, Denis Brun, Stuart J. Edelstein, Nicolas Le Novère

**Affiliations:** 1 Babraham Institute, Cambridge, United Kingdom; 2 EMBL-EBI, Hinxton, United Kingdom; 3 Amadeus IT Group, Sophia Antipolis, France; Icahn School of Medicine at Mount Sinai, UNITED STATES

## Abstract

Calmodulin is a calcium-binding protein ubiquitous in eukaryotic cells, involved in numerous calcium-regulated biological phenomena, such as synaptic plasticity, muscle contraction, cell cycle, and circadian rhythms. It exibits a characteristic dumbell shape, with two globular domains (N- and C-terminal lobe) joined by a linker region. Each lobe can take alternative conformations, affected by the binding of calcium and target proteins.

Calmodulin displays considerable functional flexibility due to its capability to bind different targets, often in a tissue-specific fashion. In various specific physiological environments (e.g. skeletal muscle, neuron dendritic spines) several targets compete for the same calmodulin pool, regulating its availability and affinity for calcium. In this work, we sought to understand the general principles underlying calmodulin modulation by different target proteins, and to account for simultaneous effects of multiple competing targets, thus enabling a more realistic simulation of calmodulin-dependent pathways. We built a mechanistic allosteric model of calmodulin, based on an hemiconcerted framework: each calmodulin lobe can exist in two conformations in thermodynamic equilibrium, with different affinities for calcium and different affinities for each target. Each lobe was allowed to switch conformation on its own. The model was parameterised and validated against experimental data from the literature. In spite of its simplicity, a two-state allosteric model was able to satisfactorily represent several sets of experiments, in particular the binding of calcium on intact and truncated calmodulin and the effect of different skMLCK peptides on calmodulin’s saturation curve. The model can also be readily extended to include multiple targets. We show that some targets stabilise the low calcium affinity T state while others stabilise the high affinity R state. Most of the effects produced by calmodulin targets can be explained as modulation of a pre-existing dynamic equilibrium between different conformations of calmodulin’s lobes, in agreement with linkage theory and MWC-type models.

## Author Summary

Calmodulin, the ubiquitous calcium-activated second messenger in eukaryotes, is an extremely versatile molecule involved in many biological processes: muscular contraction, synaptic plasticity, circadian rhythm, and cell cycle, among others. The protein is structurally organised into two globular lobes, joined by a flexible linker. Calcium modulates calmodulin activity by favoring a conformational transition of each lobe from a closed conformation to an open conformation. Most targets have a strong preference for one conformation over the other, and depending on the free calcium concentration in a cell, particular sets of targets will preferentially interact with calmodulin. In turn, targets can increase or decrease the calcium affinity of the calmodulin molecules to which they bind. Interestingly, experiments with the tryptic fragments showed that most targets have a much lower affinity for the N-lobe than for the C-lobe. Hence, the latter predominates in the formation of most calmodulin-target complexes. We showed that a relatively simple allosteric mechanism, based the classic MWC model, can capture the observed modulation of both the isolated C-lobe, and intact calmodulin, by individual targets. Moreover, our model can be naturally extended to study how the calcium affinity of a single pool of calmodulin is modulated by a mixture of competing targets *in vivo*.

## Introduction

Calmodulin is a ubiquitous calcium-binding protein involved in many cellular processes, from synaptic plasticity to muscular contraction, cell cycle regulation, and circadian rhythms. Structurally, it is organized in two highly homologous globular domains joined by a flexible linker [1]. Each domain contains two calcium-binding EF-hands that can undergo a transition between closed and open conformations, the latter favored by calcium binding [2, 3]. The transition to the open state results in exposure of hydrophobic residues able to interact with numerous binding partners [4]. However, some targets interact preferably with the closed form [5].

The shift of calmodulin’s calcium saturation curve in the presence of targets was studied experimentally by several groups [6–10]. Targets that markedly increase calmodulin’s calcium affinity include the calcium-calmodulin dependent kinase II (CaMKII), protein phosphatase 2B (PP2B) and skeletal muscle myosin light chain kinase (skMLCK). It was shown that, in general, targets do not bind calmodulin exclusively in either calcium-saturated or calcium-free forms, but rather bind to both forms with different affinities. Numerous biologically relevant targets exhibit this behavior, such as skMLCK, CaMKII, and the NaV1.2 sodium channel [7, 10–13]. The binding domains that preferably interact with calcium-free calmodulin are called IQ motifs, a family of 14-residue sequences named after the two most frequent initial amino acids (usually isoleucine, followed by a highly conserved glutamate) [5].

Calmodulin’s properties, such as cooperativity and affinity modulation by binding targets, can be explained by an MWC allosteric model. The first model of allosteric transitions was introduced in the seminal paper by Monod, Wyman and Changeux in 1965, which dealt with multimeric proteins whose subunits could undergo concerted conformational transitions, and also be modulated by target binding either to the R state, or the T state, in an exclusive fashion [14]. The model was then further extended by Rubin and Changeux to describe molecules that could be modulated by binding partners capable of binding both conformational states, but with different affinites [15]. The hypothesis of the existence of distinct calmodulin conformations in thermodynamic equilibrium, which is crucial to an allosteric model, is supported by evidence of conformational dynamics of the protein in solution, with a time constant on the order of microseconds [16–18], and was also suggested by theoretical and computational analysis of coarse-grained Hamiltonians [19, 20]. Structural studies also supported the hypothesis that a small fraction of calmodulin molecules can exist in a more compact conformation even in the presence of calcium [21].

**Figure 1:**
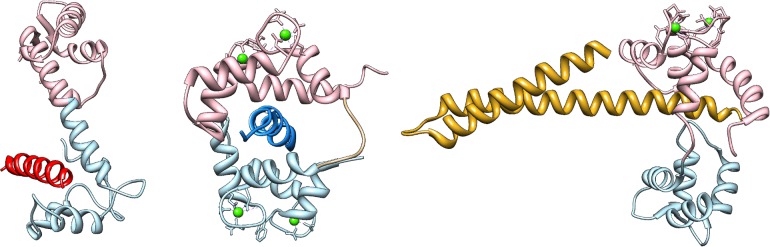
Examples of calmodulin’s capability to bind diverse targets. Calmodulin’s N-lobe and C-lobe are in pink and light blue, respectively. Calcium ions are in green. Left: calcium-free calmodulin binding to a peptide of neurogranin (red) with its C-lobe [59]. Center: calcium saturated calmodulin wrapping around a peptide of MLCK (blue) with both lobes [60]. Right: calmodulin binding to a peptide of the SK channel (yellow) via a calcium-saturated N-lobe [47]. Several studies have shown that the linker region between the two domains is inherently flexible, and can assume different conformations depending on the orientation that the globular domains require for binding [61].

Experimental studies with constrained mutants showed that the capability to switch conformation is necessary for cooperativity [3, 22, 23], which is compatible with an MWC model. Interestingly, conformational transitions are not common to all EF-hand based proteins, despite their high structural homology in calcium-free conditions. For example, calbindin D9k is structurally nearly identical to a calmodulin N-lobe, but its EF-hand remains closed after binding calcium [24]. It was proposed that the hydrophobic pockets of the open calmodulin lobes are stabilised by the unusual local abundance of the usually rare methionine residues (over 6% for calmodulin, against the 1% of the average protein) which have the highest flexibility, minimum steric hindrance and minimum solvation energy of all hydrophobic amino acids, and can therefore adapt to both segregated and and solvent-exposed conditions [4]. The flexible aliphatic side chains of methionine can also easily establish contacts with very diverse substrates, contributing to calmodulin’s promiscuity [25]. Some examples of calmodulin’s capability to bind very diverse targets is shown in Fig. 1.

**Figure 2:**
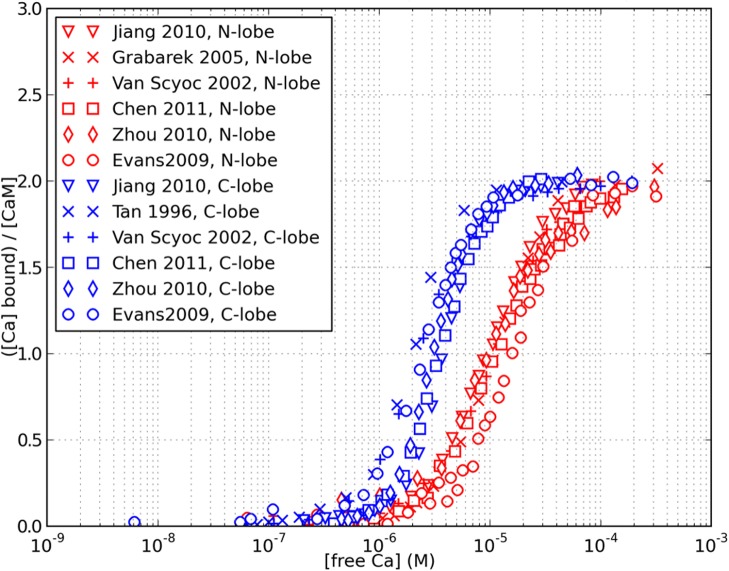
Different apparent calcium affinity of the N and C lobe. Experimental data was taken from references [3, 9, 22, 31, 62–64]

Despite their conceptual simplicity, the application of allosteric models can be challenging. The intrinsic calcium affinities of different conformational states are not easily measurable and need to be reverse-engineered, because most experimental results are fitted using the phenomenological Hill or Adair-Klotz models, which do not incorporate conformational transitions. In addition, modelling intact calmodulin, within which each domain can switch its conformation independently, results in a large number chemical species that need to be explicitly enumerated (combinatorial explosion), and a larger number of parameters that need to be fitted simultaneously. A previous allosteric model of calmodulin was developed by Stefan et al. [26], to model the differential calmodulin-dependent activation of calcineurin and CaMKII in synaptic plasticity. However, the model postulated that both lobes would undergo concerted conformational transitions, and also had similar calcium-binding properties. In fact, the two lobes possess a remarkable degree of autonomy [27], and the calcium saturation curve of calmodulin was almost exactly reproduced by superposition of the saturation curves of tryptic fragments TR1C and TR2C, containing respectively the N-terminal or C-terminal lobe only [6, 28, 29]. The four alpha-helices of each globular domains form two EF-hands that work together as one cooperative unit with two calcium-binding sites [30]. Despite of their high level of structural similarity, the two domains also exhibit strikingly different affinities and binding kinetics for calcium ions. The C-lobe has a 10-fold higher affinity, but much slower kinetics, than the N-lobe [31, 32]. The clearly different saturation curves of the lobes, as observed in the intact molecule, are shown in Fig. 2.

**Figure 3:**
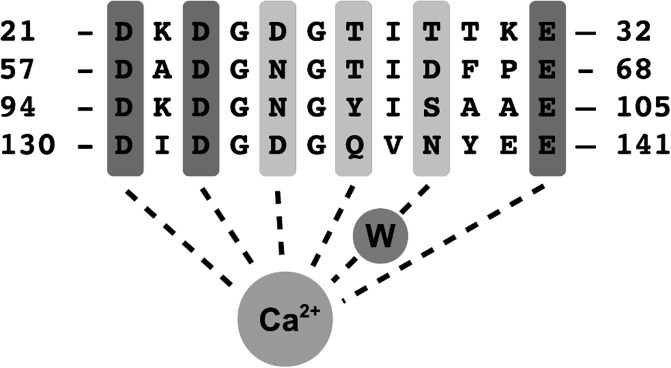
Sequence of the four calcium-binding pockets (EF-loops) of calmodulin. The calcium ion coordinates with the first, third, fifth, seventh and twelft residue of the loop, and also with the ninth through a coordinating water molecule (W). Highly conserved residues are in dark gray.

The differences in calcium-binding behavior are surprising in domains that are structurally so similar, but several studies showed that the EF-hand motif is tunable over a wide range of affinity and kinetics by modification of few key residues [33, 34]. The incredibly high level of sequence conservation across species suggests that the different properties of the two lobes are in some way crucial to calmodulin’s function. Moreover, even mutations that do not affect calcium-binding properties, but alter calmodulin’s affinity for one or more targets, can be lethal [35]. The sequence of calmodulin’s four EF-loops (12-residue calcium-binding pockets within each EF-hand) is given in Fig. 3.

The C-lobe was reported to play a pivotal role in mediating calmodulin’s calcium-dependent interactions with its targets [36], and experiments with tryptic fragments of calmodulin consistently showed that the affinity of the C-lobe for calmodulin-binding domains is usually much higher than the affinity of the N-lobe [7, 13]. Moreover, targets that bind calcium-free calmodulin seem to interact almost exclusively with the C-lobe [10, 37, 38]. In the light of these facts, we postulated that a major portion of the observed target-induced modulation effects could be explained by the interactions of the targets with the C-lobe only, and we focussed our initial effort on modelling the observed properties of TR2C, (i.e. the isolated C-lobe). Taking advantage of existing experimental data sets, we modelled the behavior of the TR2C tryptic fragment in the presence of peptides WFF and WF10 (peptides that express full-length and truncated versions of the calmodulin-binding domain of skMLCK) and Nav1.2IQp (a peptide based on the calmodulin-binding domain of the calcium-activated NaV1.2 sodium channel). We developed and parameterised a model of the tryptic fragment, in order to reliably reverse-engineer some intrinsic properties of the C-lobe of calmodulin. We then parameterised an MWC model of the isolated N-lobe by postulating that the R-states of both lobes had very similar affinities (as suggested by [6]), thus reducing the number of free parameters to estimate (see Methods).

This simplifying assumption was needed to circumvent the comparative scarcity of experimental data on the isolated N-lobe. The two submodels were then combined into a model of intact calmodulin, under the assumption that the protein behaved as the sum of its parts.

The MWC model explains cooperativity and target-induced affinity modulation as emergent properties, rather than *a priori* assumptions, as opposed for example to the Adair-Klotz model. The two formulations are however mutually consistent, and MWC and Adair-Klotz models are interconvertible, as shown by Stefan et al. [39], since for any MWC model the corresponding Adair-Klotz parameters can be computed. A notable advantage of a MWC model is that the effects of multiple competing targets are straightforward to incorporate, simply by defining each target’s affinity for the different conformational states of calmodulin. The work described in this paper shows that a carefully parameterised MWC model can indeed give reliable predictions of how individual targets modulate calmodulin affinity, and can also help investigate biologically relevant situations where numerous targets simultaneously modulate (and compete for) the same calmodulin pool.

## Results

**Figure 4:**
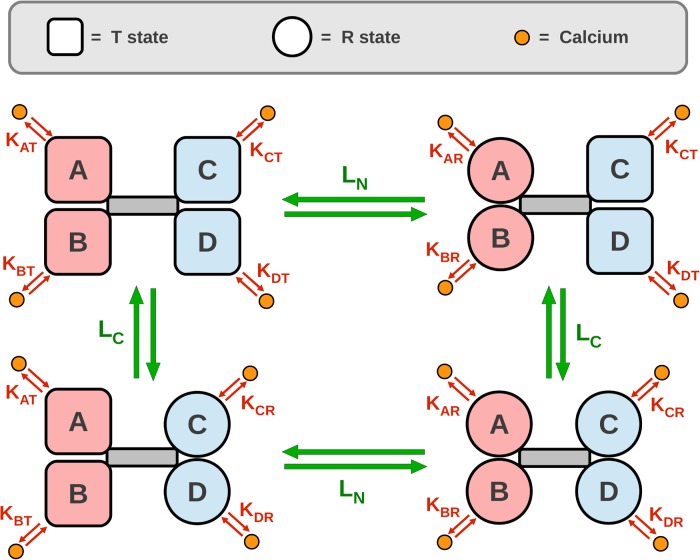
Allosteric model of calmodulin with hemiconcerted conformational transitions. Calmodulin has 4 calcium-binding sites (A, B, C, D) corresponding to one EF-hand each, and organised in two globular domains, N-lobe (pink) and C-lobe (light blue). Green arrows represent conformational transitions, red arrows represent calcium binding. Each binding site is modelled as a subunit that has access to two conformational states: open, high-affinity R state, and closed, low-affinity T state. Subunits on the same lobe are constrained to be in the same state, but the two lobes are allowed to be in different states. When no ligand is present, 4 possible configurations (TT, RT, TR, RR) coexist in thermodynamic equilibrium. A lobe that undergoes a T-to-R transition increases its affinity for calcium. Under these assumptions, it follows from classical linkage theory that ligand binding shifts the conformational equilibrium towards the open form, by stabilising the higher-affinity R state. Cooperativity is predicted as an emergent property.

A diagram of the allosteric model of calmodulin with hemiconcerted conformational transitions is shown in Fig. 4. Our model was built in three steps. First, we parameterised a model of TR2C, (isolated C-lobe), then parameterised a model of TR1C (isolated N-lobe), and finally the two submodels were merged into a model of intact calmodulin. (see Methods). The model of intact calmodulin (in SBML format) was deposited in BioModels Database [40] and assigned the identifier MODEL1405060000.

### Preliminary analysis of possible parameterisations

**Figure 5:**
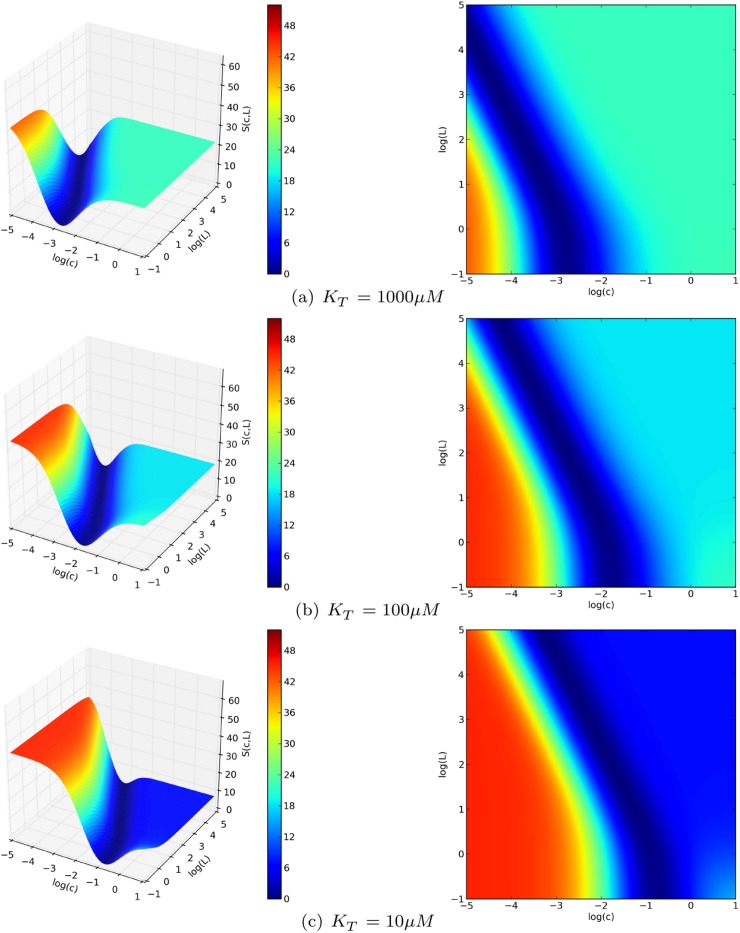
Exploratory study of the general beahviour of a two-site MWC model. The affinity of the T state was fixed at specific values within the estimated range (a: 1000μM, b: 100μM, c: 10μM) and the sum of the squared errors from the experimental points of the saturation curve, S, was plotted as function of the MWC parameters L and c. A lower score means a better fit. Over a very wide range of hypothetical, but physiologically plausible values of the T-state affinity (1 mM—10μ M), S(L, c) had the same qualitative behavior. All the pairs of (L, c) values that lie at the bottom of a flat valley (dark blue area) give a very good fit of the saturation curve of TR2C in the absence of targets. The knowledge of the target-free saturation function alone is therefore insufficient for univocal parameter identification.

The first requirement we set for our model was the capability to reproduce the saturation curve of TR2C alone. We modelled TR2C as an MWC protein with two identical binding sites and two conformational states (T and R). In the absence of targets, Equation 5 (see Methods), that expresses the fractional saturation as a function of the concentration of free intracellular calcium, simplifies to the form

$$\bar{Y}=\frac{\alpha (1+\alpha )+L\alpha c(1+\alpha c)}{{\left(1+\alpha \right)}^{2}+L{\left(1+\alpha c\right)}^{2}},$$ (1)

where:

$$L=T_{0}/R_{0}$$ (2)

is the allosteric constant in the absence of targets, defined as the ratio between the concentrations of the T and R states, when no ligand is bound),

$$\alpha ={\left[Ca_{2+}\right]}_{free}/K_{R}$$ (3)

is the ratio between the ligand affinity of the R state and the concentration of free ligand, and

$$c=K_{R}/K_{T}$$ (4)

is the ratio of the ligand affinities of the R and T state. Equation 1 depends on only 3 free parameters (L, *K*_*R*_, *K*_*T*_). We observed that the intrinsic affinity of the T state must lie within the wide, and biologically plausible, 1 μM–1 mM range. Once the value of *K*_*T*_ is chosen, the model can be rewritten as a function of the “classical” MWC parameters, L and c [14]. For any choice of *K*_*T*_, the score function of the fitting is a surface on the the (*L*, *c*) plane, with the same qualitative features, as shown in Fig. 5.

In particular, regardless of the value chosen for *K*_*T*_, all the generated fitting landscapes showed a flat valley, i.e. a region of parametric space where many different choices of L and c could all fit the saturation curve very well. Although it was technically possible to locate a point of global minimum in this region, its position was not robust to noise, and moved erratically within the valley if random errors were applied to the data sets (data not shown). This portion of parameter space contained an ensemble of possible parameter choices that lie between two limit cases: in the first one, the change of affinity upon conformational transition is relatively small (*c* ≃ 0.01), and the allosteric constant is also comparatively small(*L* ≈ 100); in the second one, the change of affinity is much greater (*c* ≤ 0.001) and so is the allosteric constant (*L* ≥ 1000). In the first case, (higher c, lower L) the molecule has a high propensity to spontaneously switch conformation to the high-affinity R state, but the R state is stabilised less strongly by the binding of calcium. In the second case (smaller c, greater L), the opposite holds true: the molecule has a very low propensity to spontaneous conformational transitions, but the stabilising effect of calcium is much stronger. It must be stressed that in both cases the model is capable of giving an excellent fit of the experimental saturation curve in target-free conditions. The knowledge of the saturation curve in the absence of targets, alone, is therefore insufficient to discriminate between the two possibilities and univocally identify the parameters. The problem could be solved using the additional information provided by the saturation curves observed in the presence of targets, which allowed us to constrain the parameter space to be sampled during the fitting procedure. The obtained constraints implied that the first of the two limit scenarios mentioned above (with greater *c* and lesser L) had to be be discarded, because the resulting model would be unable to account for the extent of the target-induced affinity shifts observed in the experiments, as explained in the Methods section.

### Truncation does not strongly affect the C-lobe’s calcium affinity

**Figure 6:**
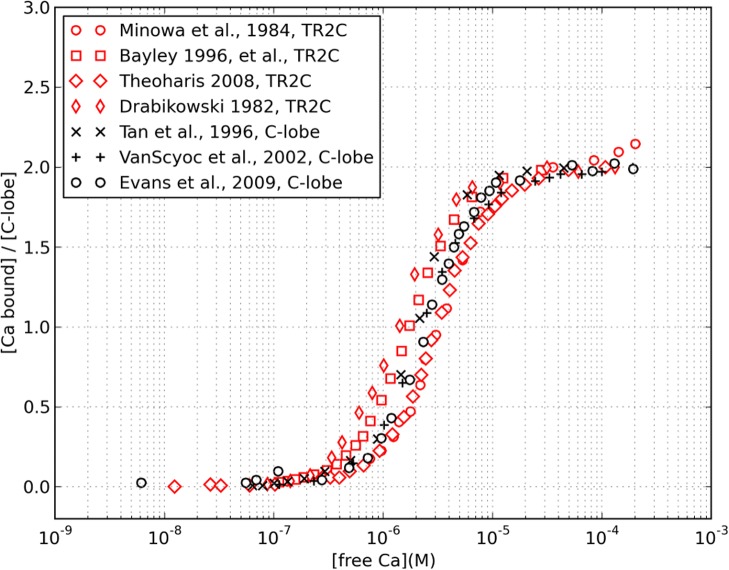
Comparison between the experimental saturation curves of TR2C and the C-lobe. Saturation of TR2C (red) and C-lobe of intact calmodulin (black), as a function of the free calcium concentration, monitored by intrinsic tyrosine fluorescence (data taken from references [7, 9, 12, 22, 28, 31, 42]). The available experimental data show some scattering, with an uncertainty of about a factor two for the concentration of free calcium necessary to produce half-saturation.

We tested whether it was legitimate to reverse engineer the intrinsic properties of the C-lobe from those of TR2C. The level of saturation of the C-lobe can be monitored in both intact calmodulin and TR2C fragments by measuring the intrinsic fluorescence of their tyrosine residues [9, 10, 12, 31, 41], It was shown that truncation does not have a strong effect on the secondary structure and tyrosine fluorescence intensity of the C-lobe [42]. In order to verify that the calcium-binding properties of the intact calmodulin’s C-lobe were maintained in the TR2C tryptic fragment, we compared the two saturation curves as presented in Fig. 6. The data shows some scattering, and the uncertainty on the calcium concentration necessary to produce half-saturation is roughly of a factor two. This is probably due to slightly different experimental conditions (see the Discussion section). However all curves were similar and shared the same qualitative behavior, meaning that truncation does not dramatically alter the lobe’s properties.

### Simultaneous fitting of saturation curves with and without peptides

The model of TR2C was parameterised by fitting the saturation curves of TR2C alone and in the presence of targets. The target ligands were chosen primarily based on the availability of published, quantitative experimental data. Also, the route we took to parameterize the model was in part dictated by the available data (see Methods). The data by Bayley and coworkers was chosen as a starting point, mainly for its thoroughness in quantitatively reporting the effects of the targets on both TR2C and intact calmodulin, both in the absence and presence of calcium [7].

**Figure 7:**
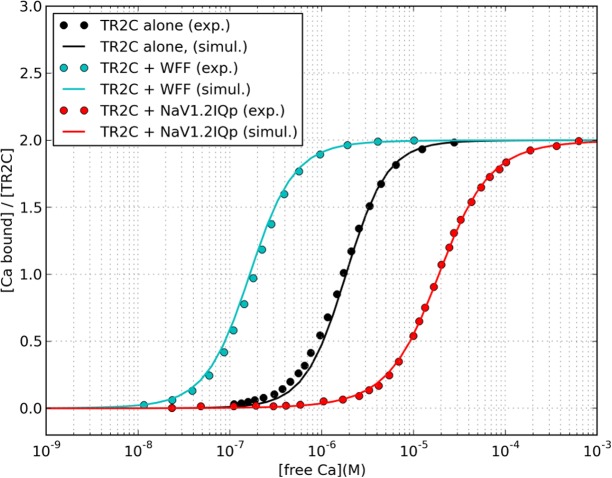
Comparison between fitted and experimental saturation curves. Experimental data was taken from references [7] and [12].

The estimated parameters are summarised in Table 1, with the resulting saturation curves shown in Fig. 7. The simulations of the model were overall in good agreement with experiments.

**Table 1:** Summary of the parameters for the model of TR2C.

Parameter	(Units)	Value	± Std
*L*	/	8.616 · 10^3^	± 4.961 · 10^3^
*K*_*T*_	M	6.241 · 10^−5^	± 5.839 · 10^−6^
*K*_*R*_	M	1.979 · 10^−8^	± 5.631 · 10^−9^
$K_{T}^{NaV}$	M	6.095 · 10^−10^	± 7.979 · 10^−11^

The reported standard deviations were automatically computed by COPASI, by inversion of the Fisher information matrix of the score function.

### Validation

**Figure 8:**
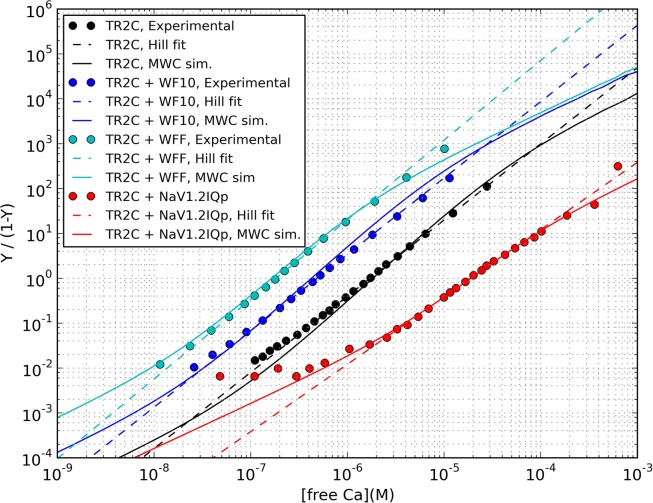
Hill plot summarising the predicted saturation curves of TR2C in the presence of WFF, WF10 and NaV1.2IQp peptides, and comparison with the artificial datasets reverse-engineered from reference [7], and actual experimental data from reference [12]. In the range of physiologically plausible calcium concentrations (from nanomolar to tens of micromolar) the MWC model (continuous lines) is close to the Hill model (dash lines).

Once the parameter values were determined, we tested the capability of the model to predict the behavior of TR2C under conditions different from those used for the fitting. The saturation curve of TR2C, as measured by Bayley and coworkers in the presence of WF10 peptide, (a truncated form of WFF with a 10-fold lower affinity for the R-state) was in good agreement with experiments (S1 Fig.). Moreover, the saturation curves by Evans and coworkers [12] were measured with two different concentrations of peptide, and it was found that doubling the concentration of Nav1.2IQp (from 1:1.4 to 1:2.8 TR2C:peptide ratio) did not produce a further shift in the saturation curve, a behavior that our model was able to reproduce (S2 Fig.). A summary of all fitted and predicted saturation curves, plotted against the corresponding experimental data points, is given in Fig. 8.

### The interplay of competing targets determines the affinity curve shift

**Figure 9:**
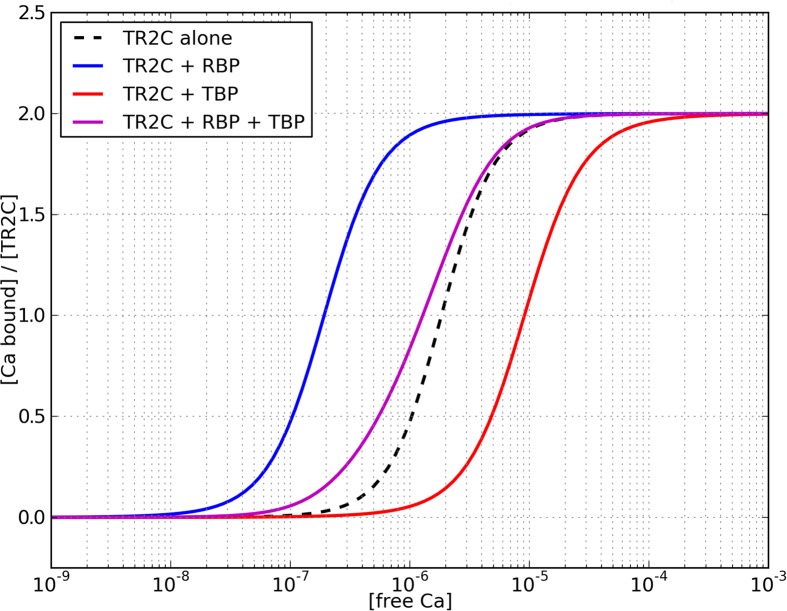
Cumulative effect of competing targets on TR2C saturation curve, in a simulated reaction chamber with 40*μM* TR2C, 40*μM* of a target with preference for the T-state (T-state binding target, TBT), and 100*μM* of a target with preference for the R state (R-state binding target, RBT). Each target alone can produce a marked shift in the saturation curve, but when both target are present in equal concentrations, the shift is much smaller. As targets, we chose peptides of two abundant neuronal proteins, neurogranin (Ng) and CaMKII, whose binding constants for TR2C were available in the literature. The chosen molar ratio (1:1:2.5) reflects estimated relative abundance of CaM, Ng and CaMKII in neuronal compartments. Their affinities for the T and R state of TR2C were respectively: 43*nM* and 1.05*μM* for neurogranin, and 88*μM* and 0.95*μM* for CaMKII [9, 59].

The high calcium affinity of the C-lobe, further enhanced by interactions within some targets (CaMKII, PP2B, skMLCK), has led to speculation that calmodulin could activate such targets even at resting calcium concentrations, i.e. in the absence of calcium signals in neuronal or muscular cells [6]. We used the model of TR2C to perform a preliminary investigation of the effect of competing targets on calmodulin. At this stage of analysis we can only investigate the part of such interactions that are mediated by the C-lobe. However, as previously mentioned, the C-lobe was shown to be mainly reponsible for mediating calmodulin-target interactions, and targets that bind the T-state with high affinity, in particular those that appear to have very little interaction with the N-lobe [36]. We simulated the steady-state response of a system containing calmodulin and two competing allosteric targets using data taken from the literature, one binding prefentially to the the T state (T-state binding target, TBT) and the other binding preferentially to the R state (R-state binding target, RBT). When both targets are present, the total saturation curve depends on their relative concentrations, as shown in Fig. 9. Shifting the concentration of targets is a potential way to tune the saturation curve of the calmodulin pool.

### Parameter estimation for the N-lobe

The model of the isolated N-lobe, in the absence of targets, was based on the model of C-lobe. The resulting parameters are summarised in Table 2. For the detailed procedure, see the Methods section.

**Table 2:** Summary of the parameters for the model of isolated N-lobe.

Parameter	(Units)	Value	± Std
*L*_*N*_	/	3.226 · 10^5^	± 2.486 · 10^5^
$K_{T}^{N}$	M	9.192 · 10^−5^	± 5.278 · 10^−5^
$K_{R}^{N}$	M	1.979 · 10^−8^	± 5.631 · 10^−9^

The R-state affinity was assumed to be equal to that of the C-lobe. The reported standard deviations were computed from the covariance matrix of the fit.

### Model of intact calmodulin

**Figure 10:**
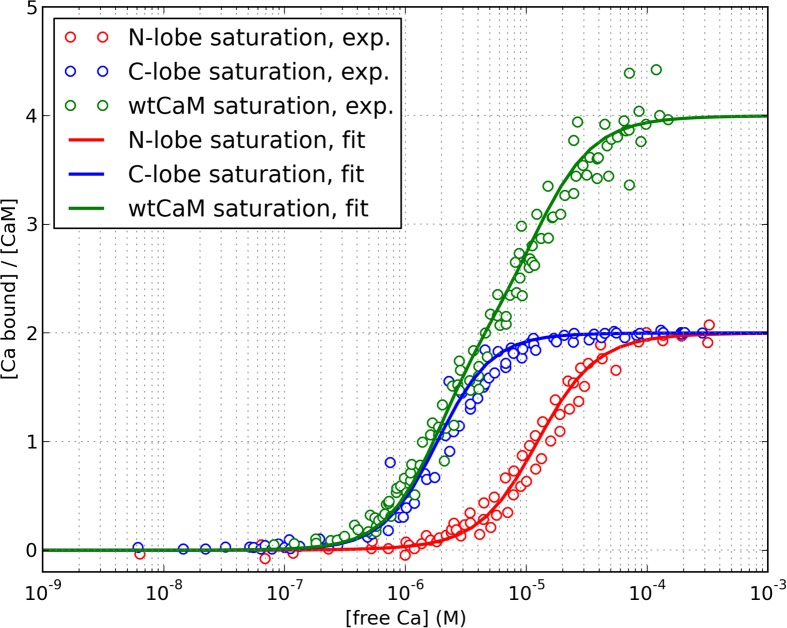
Saturation curve of individual lobes and intact calmodulin, as predicted by our model, and comparison with experimental data. The datapoints for intact calmodulin were taken from references [6, 7, 65].

The model of intact calmodulin was built by combining the two models of the N and C and lobe. The saturation curve agreed very well with the available experimental data as shown in Fig. 10. A model including calmodulin and binding targets was then automatically generated in SBML format, as described in the Methods section, and simulated in COPASI.

### Modulation of intact calmodulin by targets

**Figure 11:**
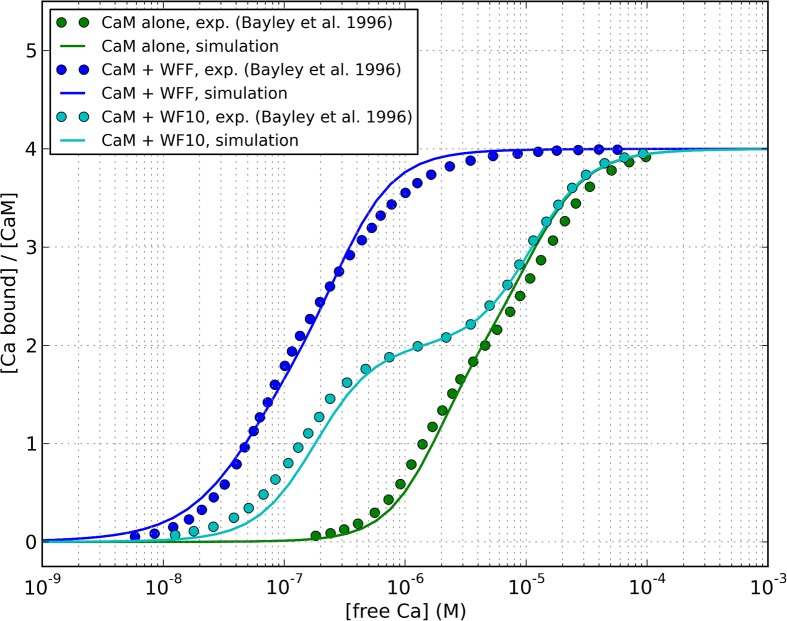
Effect of skMLCK peptides on the saturation curve of calmodulin, as predicted by our model, and comparision with experimental data by [7].

We parameterised the model by fitting it to available experimental data. Then, a posteriori, we showed that the model was consistent with experiment, because it could reproduce additional data that had not been used in the parameter estimation process, as shown in Fig. 11. The effect of a target on the saturation curve of a MWC molecule depends on how the different conformations are stabilised, which in turn depends on the affinity of the target for each conformation. The allosteric models of isolated lobes assume that each lobe can only exist in two states, T and R, which implies that both EF-hands on each lobe always undergo concerted conformational transitions. On the other hand, the model of intact calmodulin contains two lobes but does not constrain them to be the same state. As a result, one needs to take into account “asymmetric” conformations, where the two lobes are in different states. The model of intact calmodulin thus contains 4 possible states, called RR, RT, TR, TT. The first and second capital letter refer to the state of the N and C lobe, respectively. For more details, see the Methods section.

Bayley and coworkers measured calmodulin saturation in the presence of two peptides: WFF, which represent the full-length CaM-binding domain of skMLCK, and WF10, a truncated version of the same domain, corresponding to the portion that interacts with the C-lobe [7]. The saturation curve with WFF was markedly shifted to the left, while that in the presence of WF10 was biphasic.

Crucially, they were careful to measure the affinity of calmodulin and TR2C for these peptides, both in calcium and calcium-saturated conditions, which gave us an excellent initial estimate for the affinity of the peptides for the RR and TT state of intact calmodulin, and for the R and T states of TR2C. The affinity of each target for the isolated C-lobe in the R state was used as an estimate of the affinity of the target for the intact molecule in the TR state (i.e. we assumed that the target interacted with the C-lobe was much more strongly than with the N-lobe, in agreement with available experimental data). The estimated affinities for the four states are summarised in Table 3. The agreement between simulation and experiment was very satisfactory (Fig. 11).

**Table 3:** Estimated affinities of WFF and WF10 peptides for intact calmodulin.

Peptide	$K_{RR}^{t}$	$K_{RT}^{t}$	$K_{TR}^{t}$	$K_{TT}^{t}$
WFF	< 0.1*nM*	> 600*μM*	735*nM*	> 600*μM*
WF10	735*nM*	200*μM*	735*nM*	200*μM*

The affinities for asymmetric conformations of calmodulin were estimated based on assumptions and actual affinity measurements from reference [7]. The affinity of WFF for the RR state was only given as upper limit (<0.1*nM*), and was assumed to be 0.05*nM*. The affinity of both peptides for the TR state was assumed to be equal to the peptide affinity for the isolated C-lobe in the R state. In the case of WF10, which is supposed to only interact with the C-lobe, the peptide affinity for the RR and TR states were set to be equal. Each peptide’s affinity for the RT conformation (which is expected to be underpopulated in steady-state conditions and therefore play a marginal role in this analysis) was set equal to the affinity for the TT state.

## Discussion

### Estimation of the affinity of the targets for the T and R state

Throughout this work, we have assumed that the affinity of targets for the T and R state could be estimated by the affinity that the targets exhibit for calmodulin in low and high calcium conditions, respectively. The possibility that the binding of target is sufficient to cause structural change (which would invalidate our assumption) was considered but discarded based on the following published data. Several authors consistently found that peptides of calmodulin targets bind calmodulin with different affinities in the absence of calcium, or at saturating calcium concentrations [7, 9, 10, 12]. The fact that calcium induces a conformational transition in calmodulin is also known from experimental results now widely accepted in the field [2, 43, 44]. Targets that bind calmodulin with high affinity in the presence of calcium generally bind the calcium-free form with a measurable but much lower (10 to 100-fold) affinity, as shown by numerous experiments [7, 9, 10]. This strongly suggests that calcium ions are actually the main determinants in driving conformational transitions. We must therefore conclude that the binding of a target alone is insufficient to deterministically produce a conformational transition to the high-affinity form (induced fit). Consequently, in the absence of calcium, targets are interacting with calmodulin molecules that are predominantly in their calcium-free form. Conversely, at the high concentrations of calcium, targets are interacting predominantly with calmodulin molecules in their calcium-bound form. In the absence of direct experimental evidence, such considerations are of course qualitative and must be regarded as assumptions. On the other hand, more dramatic peptide-induced effects, such as folding of previously unstructured portions of the protein, can probably be safely ruled out, given structural evidence that calmodulin is a well-folded protein over a wide range of calcium concentrations, and that its conformational transitions mostly occurs by rearrangement of well-folded secondary structural elements [45, 46]. Moreover, our model explicitly contemplates the possibility of conformational transitions for calmodulin bound to a peptide but not to calcium (see Methods). Whether this T to R transition in the peptide-bound state is triggered by the presence of target peptide, or whether the R state is merely stabilised by the peptide after a transition produced by spontaneous thermodynamic fluctuations, is a very subtle question. The latter scenario however still provides a simple and internally coherent mechanistic explanation.

### Assumption of hemiconcerted transitions and lobe independence

The work presented in this article deviated from a model previously published by our group, because the conformational transitions of calmodulin’s lobes are no longer assumed to be fully concerted. Concerted transitions were an assumption that greatly simplified the resulting model but, we later found, also conflicted with a published experimental paper, which gave evidence that asymmetric conformational states are possible [26, 47]. Moreover, several authors had previously argued in favour of lobe independence or very high degree of autonomy [28, 29, 48, 49]. Lobe independence also offer a natural explanation as to why targets that interact with only one lobe (such as the WF10 peptide by Bayley et al.) seem to only modulate half of the saturation curve. A fully concerted model would not be suitable, for example, to incorporate the effects of an important neuronal protein such as neurogranin, which mostly interacts only with the C-lobe of calmodulin [37]. Even if the conformational transitions were not fully concerted, there could be a measure of coupling between lobes, i.e. the state of one lobe could affect the probability of conformational transition of the other. Much of our parameter estimation work rests upon the assumption that there is no evidence of strong coupling effect. As for the possibility of weak coupling, there is no clear consensus in the literature. If coupling effects were strong between the lobes, the behaviour of the tryptic fragments containing one calmodulin lobe would show evident divergence from the behaviour of the lobes in the intact protein (which can be monitored separately as shown by [31]). However, several experimental studies show that lobes of intact calmodulin, and tryptic fragments, have very similar calcium binding properties [3, 7, 28, 29]. Given the scattering of experimental data, weak coupling effects, with a magnitude smaller than the noise level, cannot be ruled out with absolute certainty, but including them would have complicated the model with no visible benefit, and therefore we decided to omit them. The two lobes may not be absolutely independent, but they clearly enjoy a remarkable degree of autonomy, and most definitely they are not rigidly coupled. There is also experimental evidence that the actual “cooperative” unit in the EF-hand protein family is the four-helix domain (i.e. in the case of calmodulin, the globular domain rather than the whole molecule) [4, 30]. The lack of lobe coupling, with respect to the conformation selection, implies that the early saturation of the high-affinity C-lobe at lower calcium concentrations does not promote an earlier transition of the low-affinity N-lobe to the R-state. If such coupling were present, the activation of the N-lobe in the intact protein would be “helped” by the higher-affinity C-lobe. The N-lobe in the intact protein would therefore show higher affinity than the isolated N-lobe. Experimental evidence, however, shows that this is not the case, at least within the level of precision allowed by the noise on the experimental data [3, 28].

### Calmodulin modulation by individual targets

We have shown that allosteric regulation can explain the modulation of the calcium-binding properties of the TR2C fragment (and hence, of the C-lobe of calmodulin) by several calmodulin-binding peptides. The immediate consequence is that for a given concentration of target, the parameter that determines the extent of the saturation curve shift, at steady-state, is the ratio of the affinities of the target for the R and T states of calmodulin. Plotting the experimental points against the fitted curves in the Hill plane also offer an explanation as to why the MWC and Hill models can both provide a very good fit, despite predicting two quite different qualitative behaviors: in the Hill plane, the Hill curve is a straight line, whilst the MWC model predicts a gradual shift from a lower to a higher asymptote. As shown in Fig. 8, in the range of physiologically plausible calcium concentrations (from nanomolar to tens of micromolar) the MWC model is close to the Hill model. The Hill coefficient predicted by the MWC model also decreases in the presence of targets, in qualitative agreement with experiments (Table 4). The simulations of intact calmodulin in the presence of WF10 shows that biphasic saturation curves can be produced by targets that only stabilise the high-affinity form of one lobe. WF10 is an artificial peptide, but targets that induce differential modulation of calmodulin domains can be found in nature, as in the case of the anthrax edema factor [50].

**Table 4:** Summary of Hill coefficients for TR2C in the absence and presence of targets.

Case	*n*_*H*_ (MWC)	*n*_*H*_ (Hill)
TR2C	1.91	1.71
TR2C + WF10	1.89	1.70
TR2C + WFF	1.77	1.77
TR2C + NaV1.2IQp	1.53	1.50

Values were calculated as the maximum slope of the Hill plot of the MWC model (left column) or from fitting the experimental saturation curve with a Hill equation (right column).

An allosteric model of calmodulin was previously developed in our group that could satisfactorily reproduce the dose-response curve of wild-type calmodulin and also explain differential activation of PP2B and CaMKII during synaptic tetanic stimulation [26]. The previous model was however based on different premises and some additional simplifying hypotheses, such as concerted conformational transitions of both lobes, and exclusive binding of the target by one conformation. A direct comparison would therefore not be meaningful.

### Calmodulin modulation by competing targets

Calmodulin *in vivo* is always in the presence of a large number of targets, which simultaneously modulate its activity. We used our model to investigate the effect of mixtures of competing targets on calmodulin’s C-lobe. We exploited the seemingly predominant role of the C-lobe in mediating calmodulin-target interactions to test the behavior of our model in the simultaneous presence of two different targets that had higher affinity for the T state and the R state, respectively. As an example scenario we chose peptides of abundant neuronal proteins, whose binding affinities for TR2C were available in the literature. We chose a 1:1:2.5 molar ratio for the three proteins, (a scenario where the concentration of calmodulin-binding proteins is much higher than the concentration of calmodulin [51]), to account for the higher concentration of targets that favour the R-state. In the chosen conditions, the effect of the competing targets was almost cancelled out and the resulting calcium saturation curve was close to that in the absence of targets, as shown in Fig. 9. Regulating the relative abundance of targets can therefore bidirectionally tune the amount of calcium bound to calmodulin’s C-lobe. At any given concentration, the target with the higher affinity is expected to exert the stronger effect.

### Two-state approximation and parameter estimation

A two-state model is of course a simplification of something as complex as a protein’s conformational dynamics. It is more plausible that calmodulin is capable of sampling a wider ensemble of conformations, and its high conformational plasticity allows it to regulate downstream protein targets that are structurally very diverse [10, 25, 52]. Moreover, the PEP-19 peptide, expressed in the cerebellum, was shown to regulate mostly calmodulin’s calcium binding kinetics, with little effect on affinity, and could do so even when calmodulin was already associated to CaMKII [53]. These observations imply that the tuning of calmodulin’s affinity and kinetics is highly adaptable, but capturing every possible feature of this mechanism was outside the purpose of the present work. For example, our model in its present form doesn’t allow calmodulin to bind more than one target, which is true for many targets but not for PEP-19 [53], but our primary focus was to present a putative mechanism to explain how a rather large class of targets modulate calmodulin’s affinity, as shown for example by [6]. For the conditions focussed on here, the agreement of a two-state model with experimental results was already satisfactory, and the regulatory properties of the above mentioned PEP-19 peptide (which is cerebellum-specific) seem to be the exception rather than the rule [53]. Therefore, the model in its present form should be applicable to a quite wide range of biological scenarios. On a technical level, parameter estimation is made challenging by the uncertainties and the scattering shown by the available experimental data, but the differences in experimental protocols do not seem so severe to invalidate the interpretation of the results (see S1 Table). Thanks to the fact that calmodulin is perfectly conserved across all known mammals, protein sequences were most definitely identical, and did not represent a potential source of uncertainty in the experimental results. In fact, for reasons of consistency, a substantial amount of published data on paramecium calmodulin, which is only 88% identical to the mammal one [10, 13], was not included in the datasets used in this work. Also, we were reassured by the fact that the vast majority of the reported data was obtained in very similar conditions with regard to pH, temperature, and ionic strength. The most significant difference in the buffer types was the amount of magnesium added to the solution, which varied from 0 to 1mM (see S1 Table). The effects of buffer conditions on calmodulin were reviewed by Ogawa and Tanokura [54], which found that pH and temperature had a measurable but weak effect on affinity and cooperativity. The uniform levels of added salt were relevant because higher ionic strength makes calcium affinity largely independent of pH within the range 6.5–8, as noted by [29], and lowers the effect of added magnesium on calcium affinity of calmodulin [54]. As discussed by [55], the main role of magnesium is most likely the stabilization of the calcium-free form, possibly to prevent proteolytic cleavage, which should not be a problem *in vitro*.

**Figure 12:**
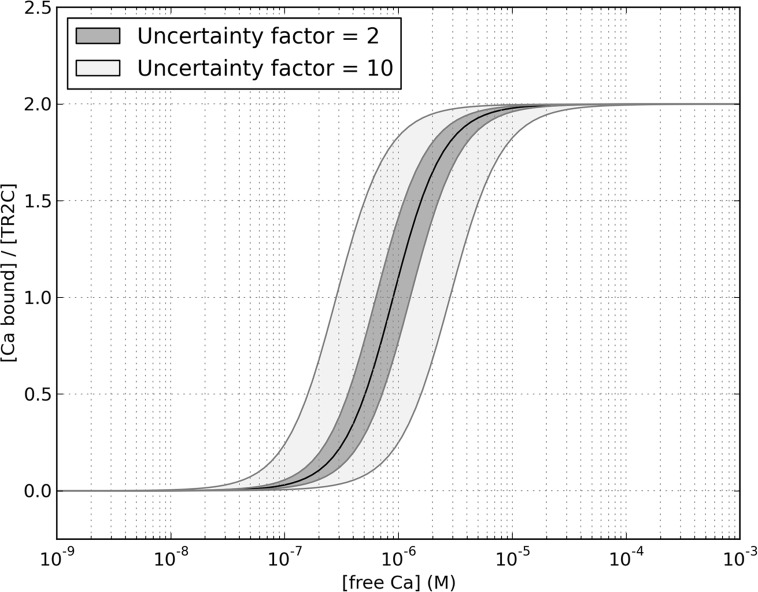
Sensitivity of the saturation curve to the value of the allosteric constant. The black line is the fitted function, the grey area shows the region that the curve would span if the allosteric constant was lowered (left shift) or increased (right shift) by a factor two (dark gray) or 10 (light gray).

Ideally, allosteric models would have to be parametrised using data sets produced in highly standardised conditions, designed to be as close to the *in vivo* conditions as possible. However, different groups performed similar experiments and measured apparent calcium affinities that sometimes differ by up to a factor of two or more. This could be due to a number of reasons, including variable levels of purity of reagents or proteins. As a consequence, pinpointing the exact value of some parameters can be challenging. However, imprecisions in the estimation of parameters do not invalidate the general behaviour of our model. For example, the standard deviation on the fitted value of the allosteric constant is rather large. On the other hand, this is mostly due to the low sensitivity of the saturation function with respect to this parameter: even an estimation error of a factor of two (which is still relatively small, and close to the noise level) would shift the saturation curve by an amount that is comparable to the scattering in the available literature data, as shown in Fig. 12.

The main goal of our mechanistic model was to test the consistency of a plausible physical mechanism with respect to the experimental observations, rather than to achieve a “perfect fit”. To justify this approach, we must first acknowledge that there exist two main types of models. The first type comprises what we call phenomenological models, which fit an analytical curve to the data points, using as many parameters as necessary to represent the data within defined limits. Such models can fit the data well, but do not necessarily provide insights into the underlying physical mechanism. The second type is that of what we call mechanistic models, where we make reasonable assumptions about a (possibly simplified) molecular mechanism that underlies the observed behaviours. A mechanistic model must abide the restriction that its parameters must have well defined physical meaning, associated with an elementary physical phenomenon. A mechanistic models will almost always give a poorer fit, but its contribution to knowledge lies in its explanatory character whereby observed complex behaviour can emerge from simpler physical principles. Given the above mentioned limitations in the precision of the available data, our message is that a major portion of the observed properties of calmodulin can be explained by a model of the second type, with the limitation that at the present state of experimental knowledge, a more precise determination of parameter values is not possible.

### Conclusions

We have shown that a classic MWC allosteric model can successfully fit the saturation curves of calmodulin lobes and of the intact protein, and also account for the effects of several biologically relevant targets. This wide applicability was also achieved with a comparative economy of hypotheses (independence of two domains, each in thermodynamic equilibrium between two conformations that have different affinities for the ligand and also different affinities for each target), thus providing a useful conceptual framework upon which further modelling work can be constructed. A crucial point is that the apparent affinity of calmodulin is modulated by simultaneous dynamic equilibria with different targets that exhibit a preference (tighter binding) to one of the possible conformations of calmodulin.

## Methods

### MWC model of an isolated calmodulin lobe modulated by targets

**Figure 13:**
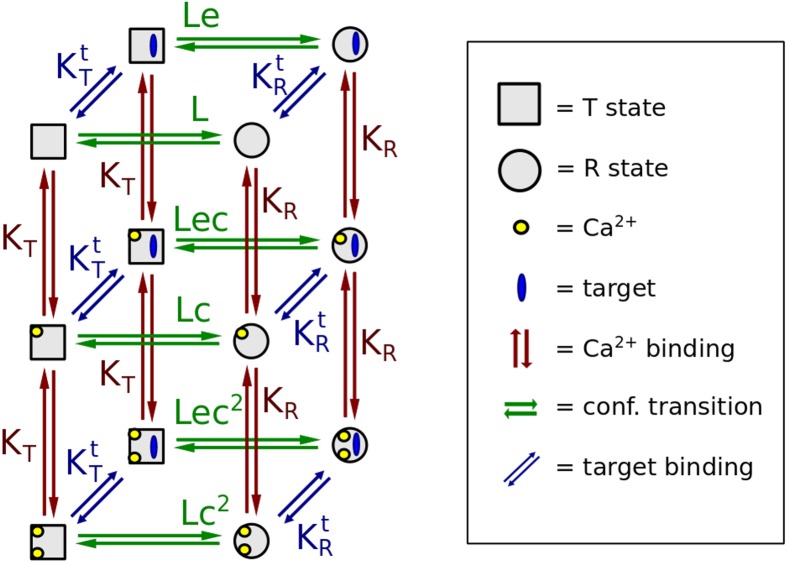
Diagram of the model used to represent isolated lobes of calmodulin (TR1C and TR2C). Each lobe has two binding sites for calcium, two possible conformations, and can bind targets with different affinities depending on its conformation. As a simplifying assumption, the two calcium-binding sites were assumed to have identical affinities. The molecule can exist in two conformational states, T and R. In the absence of ligand, the equilibrium constant for the R to T transition is the allosteric constant L. The R state has affinity *K*_*R*_ for calcium and $K_{R}^{t}$ for target *t*. The T state has affinity *K*_*T*_ for calcium and $K_{T}^{t}$ for target *t*. In accordance to classic linkage theory, each calcium ion bound to the molecule shifts the conformational equilibrium towards the state with the higher calcium affinity, causing a scaling of the allosteric constant L by a factor *c* = *K*_*R*_/*K*_*T*_. In an analogous fashion, the binding of a target shifts the conformational equilibrium towards the state that binds *t* with the higher affinity, and scales L by a factor $e=K_{R}^{t}/K_{T}^{t}$. The model of intact calmodulin was obtained by combining two of these sub-models.

Each isolated lobe of calmodulin exhibits cooperativity between its two calcium binding sites. We chose to model the isolated C-lobe (TR2C tryptic fragment) as a two-subunit, two-state MWC molecule, where the subunits undergo concerted conformational transitions. As a starting point, we assumed that both calcium-binding sites on the C-lobe were identical, as was observed for example for the CaM85/112 mutant [22]. Therefore, in our model, both sites have calcium affinity *K*_*T*_ when they are in the T state, and a higher affinity *K*_*R*_ when the lobe is in the R state. Targets can bind both conformations of the molecule, but with different affinities, as shown by several experimental studies [7, 12]. A diagram of the resulting model is given in Fig. 13.

Under the simplifying assumption, used throughout this work, that the two calcium-binding sites are identical, the following equations can be derived for the saturation function of TR2C in the presence of an allosteric target A [15]:

$$\bar{Y}=\frac{\alpha \left(1+\alpha \right)+L^{\prime }\alpha c\left(1+\alpha c\right)}{{\left(1+\alpha \right)}^{2}+L^{\prime }{\left(1+\alpha c\right)}^{2}}$$ (5)

$$L^{\prime }=L{\left(\frac{1+\gamma e}{1+\gamma }\right)}^{2}$$ (6)

where:

$$L=T_{0}/R_{0}$$ (7)

is the allosteric constant in the absence of targets (defined as the ratio between the concentrations of the T and R states, when no ligand is bound), *L*′ is the allosteric constant in the presence of targets,

$$\alpha ={\left[X\right]}_{free}/K_{R}$$ (8)

is the ratio between the ligand affinity of the R state and the concentration of free ligand,

$$c=K_{R}/K_{T}$$ (9)

is the ratio of the ligand affinities of the R and T state,

$$\gamma ={\left[A\right]}_{free}/K_{R}^{t}$$ (10)

is the ratio between the concentration of free allosteric target, [*A*]_*free*_ and the target affinity of the R state, $K_{R}^{t}$, and

$$e=K_{R}^{t}/K_{T}^{t}$$ (11)

is the ratio between the target affinity for the R state $K_{R}^{t}$, and that for the T state $K_{T}^{t}$. The above equations allow the calculation of the saturation level of TR2C in the presence of a known concentrations of *free* ligand (calcium) and allosteric targets (peptides). They also clearly show that the effect of targets is equivalent to a modulation of the allosteric constant, whilst the other parameters remain unaffected. The function $\bar{Y}$ describes a surface in the plane (*α*,*γ*), as shown in Fig. 14. The ligand saturation of an allosteric molecule, at equilibrium and at a given concentration of free ligand and free target, is entirely determined by the following parameters (note that all affinities are expressed as *dissociation* constants):

**Figure 14:**
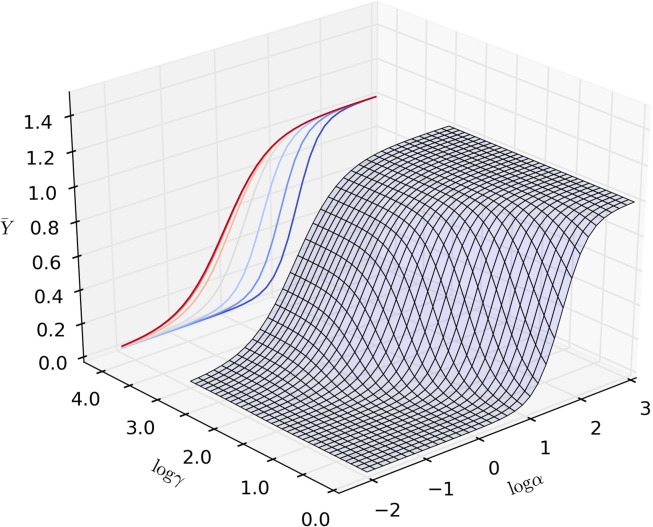
Saturation function of an MWC allosteric model of a two-state molecule with 2 binding sites, such as TR2C, in the presence of a target with preference for the R state. Increasing concentrations of target produce a left shift in the calcium saturation curve.

- the allosteric constant *L* (or isomerisation constant), defined as the concentration ratio between the R and T states in the absence of ligand.
- the ligand affinity of the binding sites in the T state, *K*_*T*_;
- the affinity change upon transition from T to R state, *c* = *K*_*R*_/*K*_*T*_;
- the affinity for the target when the molecule is in the T state, $K_{T}^{t}$;
- the change of affinity for the target upon transition from the T state to the R state, $e=K_{R}^{t}/K_{T}^{t}$.

A diagram of an MWC model of TR2C is given in Fig. 13. However, most available experimental data were obtained performing calcium titrations of calmodulin the presence of a fixed *total* concentration of target, and therefore Equation 5 was not directly applicable for fitting purposes. This apparent difficulty can be circumvented analytically, by calculating the corresponding concentrations of free target, as shown for example by Martinez et al. [56]. However, we found convenient to follow a computational route, directly implementing the model in the biochemical pathway simulator COPASI, which supports parameter fitting of steady-state properties when a system is initialised with total concentrations of reagents in a reaction compartment. The analytical model was instead used for a preliminary study on the general behavior of an MWC model for several choices of parameters, and also to discriminate between alternative possible parameterisations, as shown in the Results section.

### Choice of experimental data for parameter fitting and validation

Several published steady-state titration curves described how calmodulin and TR2C fractional saturation at varying concentrations of free calcium change in the presence of different peptides that mimic the consensus motif of several *in vivo* binding partners of calmodulin. Published datasets were digitised using the freely available PlotDigitizer program. The datasets of Bayley et al. [7] and Theoharis et al. [12] were of particular interest because they reported the affinity for the peptides both in the absence and at saturating concentrations of calcium. In an allosteric perspective, these data provide estimates of the intrinsic affinity of the peptide for the T-state (predominant in calcium-free calmodulin) and R-state (predominant in calcium-saturated calmodulin). These affinities were thus known quantities in our model. The properties of the peptides used in the above mentioned experiments are summarised in Table 5.

**Table 5:** Summary of the peptides used to fit the computational model.

Peptide	Description	$K_{T}^{t}$	$K_{R}^{t}$	Source
WFF	Full-lenght CaM-binding domain of skMLCK	1.6*μM*	76*nM*	[7]
WF10	Truncated CaM-binding domain of skMLCK	1.1*μM*	712*nM*	[7]
NaV1.2IQp	IQ domain of the NaV1.2 sodium channel	≤ 25*nM*	≤ 75*nM*	[12]

The superscript *t* indicates calmodulin’s affinity for a target.

It must be noted that none of the experimental datasets in the literature reported error bars or standard deviations for the measured datapoints, even when the datapoints were averages of measurements performed in triplicate [12]. In reality, all plotted datapoints are affected by uncertainty both on the x (free calcium concentration) and y axis (saturation) although free calcium concentration is usually known to high precision [6]. We estimated the TR2C model parameters by fitting experimental calcium saturation curves of TR2C alone, TR2C+WFF peptide (1:1.4 molar ratio), TR2C+Nav1.2IQp peptide (1:1.4 molar ratio). A fourth and fifth data set, TR2C+WF10 (1:1.4 molar ratio) and TR2C+Nav1.2IQp peptide (1:2.8 molar ratio) were not used for parameter fitting and were kept for validation purposes. Data sets were taken from references [7, 12].

### Fitting procedure and choice of constraints on the parameter space

The resulting computational model had many parameters but, crucially, the MWC equations constrained the majority of them to be known functions of a a much smaller number of free parameters. These free parameters were the only ones that needed to be fitted. In the absence of targets, the COPASI model contains 3 independent parameters, (L,*K*_*R*_,*K*_*T*_). In the presence of target *t*, the saturation curve depends also on the target affinities $K_{R}^{t}$, $K_{T}^{t}$, which were both known quantities for WFF. The affinities of the peptide NaV1.2IQp for both the T and R states were in the nanomolar range, and only estimated upper limits for their value were available in the literature (Table 5). We assumed that NaV1.2IQp affinity for the R state was equal to the estimated upper limit, while its affinity for the T state was set as an additional parameter to be fit, which we called $K_{T}^{NaV}$. In total, the 4 independent parameters *K*_*T*_, *K*_*R*_, $K_{T}^{NaV}$ and *L* were required to fit simultaneously the three experimental data sets, i.e. the score function minimised in the fitting was the total sum of square residues from all three available datasets. The contributions of each dataset to the total sum of square residues were weighted to account for the fact that not all datasets contained the same number of data points. When choosing the initial conditions for the fitting procedure we assumed that our model must account for a wide range of observed behaviors. An initial exploratory study on different possible parameterizations was performed using the analytical MWC model described in the previous paragraphs, using custom Python scripts. These preliminary results were used to formulate additional constraints on the parametric space that the computational model was allowed to sample, when fitted to the experimental data describing the saturation curve of TR2C in the presence of targets. In the MWC framework, the apparent ligand affinity is produced by a mixture of two distinct populations of molecules, in the R-state and T-state. Target peptides shift the saturation curve by differentially stabilising the R and T state. Therefore, any observed saturation curve, with or without targets, is bound to lie between two limit curves corresponding to populations of 100% R state, and 100% T state. The two fully-stabilised states also exhibit no cooperativity, because no further population shift is possible. These observations provide a simple way to determine lower bounds for *K*_*T*_ and upper bounds for *K*_*R*_, which in turn provide an upper bound for *c* = *K*_*R*_/*K*_*T*_. The saturation curve for TR2C in the presence of Nav1.2IQp showed an apparent affinity of about 10*μM* and a Hill coefficient greater than 1, indicating that the T-state was not fully stabilised [12]. Therefore the affinity of the pure T-state must be lower than 10*μM*. On the other hand, data obtained with full-length calmodulin, showed that some peptides can increase affinity and decrease cooperativity, shifting the saturation curve to the left to an extent that implies that the calcium affinity of the pure R state must to be higher than 20nM [6], assuming that the C-lobe is responsible for the higher affinity when CaM is bound to a target, as shown by data published by Shea et al. [9]. Taken together, these observations led to the following constraints on the allowed parameter values:

$$K_{R}\leq 20nM$$ (12)

$$K_{T}\geq 10\mu M$$ (13)

$$c=\frac{K_{R}}{K_{T}}\leq 0.002.$$ (14)

The fitting was performed using the built-in parameter estimation functions of COPASI, using 1000 iteration of a genetic algorithm with stochastic ranking [57], with a population size of 20, and with the constraints on the parameter values described in the previous paragraph. A genetic algorithm is a non-local optimisation method and is therefore less prone to converging to local minima in comparision to gradient-based methods.

### Calculation of Hill coefficients

The Hill coefficients of calmodulin and calmodulin in the presence of targets were calculated as described in Ref. [58], as the slope of the saturation function in the Hill plane:

$$n_{H}=\frac{d\left(\log\frac{\bar{Y}}{1-\bar{Y}}\right)}{d\left(\log\alpha \right)}$$ (15)

where *α* = [*Ca* ^2+^]_*free*_/*K*_*R*_ is the free ligand concentration normalised by the ligand affinity of the R state, and the fractional saturation function, $\bar{Y}$, is defined as the ration between the number of occupied binding sites and the total number of binding sites, at a given concentration of free ligand, and can be calculated as:

$$\bar{Y}=\frac{{\left[Ca^{2+}\right]}_{bound}}{2\cdot [TR2C]}$$ (16)

For an MWC model with two identical subunits, the Hill coefficient as a function of ligand concentration describes a symmetric bell-shaped curve, with its maximum at the point of half-saturation.

### Parameterization of isolated N-lobe

The model of isolated N-lobe (corresponding to the TR1C tryptic fragment) was formally identical to the TR2C fragment described in the previous paragraphs. However, the parameter estimation required a different approach. We could not directly fit the model to saturation curves in the absence and presence of targets, because the affinity of the isolated N-lobe for targets is very low [7], and the resulting shift of the saturation curve very weak [12]. Moreover, targets that bind calmodulin in calcium-free conditions have little or no interaction with the N-lobe, and their effect on its saturation curve was negligible. First we assumed, for the sake of simplicity, that the two calcium-binding sites of the N-lobe are identical (as we did for the C-lobe). Based on the experimental evidence [6], that the two lobes have very similar affinity when bound to high-affinity targets, we made the additional assumption that, when in the R state, both lobes had the same calcium affinity. The affinity of the C-lobe in the R state was already known from the parameterization of the TR2C model. We estimated the calcium affinity of the N-lobe in the T state by fitting the saturation curve by Grabarek and coworkers for the NCaM41/75 mutant (where the N-lobe was constrained in a closed conformation by a disulfide bond) with a non-cooperative model with two identical binding sites. Knowing the affinity of both the R and T state, the allosteric parameter *c* = *K*_*R*_/*K*_*T*_ was readily calculated. The only remaining free parameter in the model was the allosteric constant L, which was estimated by fitting and analytical MWC model onto the available experimental points for the saturation curve of the N-lobe [3, 9, 31].

### Model of intact calmodulin

#### Generation of the model in SBML format

The model of intact calmodulin was built by assuming that each lobe still behaved as it would in its isolated form. When referring to the conformation of intact calmodulin, we adopt the following convention: “RT” means that the N lobe is in the R state, and the C-lobe is in the T state. Since the two lobes have different affinities and therefore saturate at different calcium concentrations, asymmetric states are biologically plausible. However, the resulting kinetic model is much more complex than the submodels comprising only one lobe. Two largely independent lobes imply that the conformations are four: RR, RT, TR, TT. The binding sites are also 4 (A, B on the N lobe and C, D on the C-lobe), which leads to 64 possible states for calmodulin only. With the addition of targets, combinatorial explosion implies that the number of equations in the kinetic model quickly increases to more than one thousand. Manual modification of such models and inclusion of additional targets would be error-prone and labour-intensive. On the other hand, the interaction rules underlying the model are simple, and the complexity is purely combinatorial, which means the model lends itself to be generated iteratively. Taking advantage of the existing libSBML library, we wrote custom Python scripts to automatically generate allosteric models of calmodulin in the presence of targets, in the SBML format. SBML is natively supported by COPASI, thus making the setup process of different kinetic models both faster and more reliable.

#### Affinity of targets for asymmetric conformations of calmodulin

Our model allows for hemiconcerted conformational transitions (i.e. the two lobes can have different conformations, but the EF-hands on the same lobe are always in the same state). This poses no problem for calcium binding events, because calcium only binds to individual sites, whose affinity is determined by their state. Targets, on the other hand, being bigger molecules (either peptides of whole proteins) bind to the whole molecule and usually interact with both lobes at once. Their affinity for a given conformational state of calmodulin can be expected to be an interplay of their affinity for the two lobes. A modelling challenge is posed by the fact that in the available experimental data, the affinity of calmodulin for a given target was usually measured only in two limit cases, in the absence of calcium (when almost all calmodulin will be in the TT state) and at saturating concentrations of calcium (when calmodulin will be mostly in the RR state). In our model we also needed the affinities for the two asymmetric states RT and TR. To overcome this limitations, we observed that targets that bind preferentially the calcium-saturated calmodulin exhibit a much stronger interaction with the C-lobe than with the N-lobe [9, 42] and that the N-lobe alone can only bind very weakly to targets, and the affinity is in the millimolar range in the absence of calcium [7, 12]. Collectively, these observations led to the following simplifying assumptions:

- The affinity of the target for the RR state is equal to the affinity for CaM, measured at saturating calcium concentrations;
- The affinity of the target for the TT state is equal to the affinity for CaM, measured in the absence of calcium.
- The affinity of the target for the TR state is equal to the affinity for the TR2C fragment (isolated C lobe) at saturating calcium concentrations.
- The affinity of the target for the RT state is roughly equal to the affinity for the TT state

With this set of assumptions, the model could reproduce several experimental saturation curves (see Results).

## Supporting Information

S1 Fig Prediction of the behaviour of TR2C in the presence of the WFF peptide. (TIF)

S2 Fig Prediction of the behavior of TR2C in the presence of the NaV1.2IQp peptide. (TIF)

S1 Table Summary of the experimental conditions under which calcium titrations were performed. (PDF)

S2 Table Summary of the parameters for the computational model of TR2C. (PDF)

S3 Table Summary of the chemical species in the TR2C model. The model for the N-lobe is formally analogous to that of TR2C. (PDF)

S4 Table Summary of the reactions used in the TR2C model. (PDF)

S5 Table Summary of the independent parameters for the model of intact calmodulin. (PDF)

## References

[1] Faga LA, Sorensen BR, VanScyoc WS, Shea MA (2003) Basic interdomain boundary residues in calmodulin decrease calcium affinity of sites I and II by stabilizing helix-helix interactions. Proteins: Struct, Funct, Bioinf 50: 381–91. 10.1002/prot.10281 12557181

[2] Zhang M, Tanaka T, Ikura M (1995) Calcium-induced conformational transition revealed by the solution structure of apo calmodulin. Nat Struct Mol Biol 2: 758–767. 10.1038/nsb0995-758 7552747

[3] Grabarek Z (2005) Structure of a trapped intermediate of calmodulin: calcium regulation of EF-hand proteins from a new perspective. J Mol Biol 346: 1351–66. 10.1016/j.jmb.2005.01.004 15713486

[4] Nelson MR, Chazin WJ (1998) Conformational changes in Ca2+ sensor proteins. Protein Sci 7: 270–282. 10.1002/pro.5560070206 9521102 PMC2143906

[5] Rhoads R (1997) Sequence motifs for calmodulin recognition. FASEB J 11: 331–340. 9141499 10.1096/fasebj.11.5.9141499

[6] Peersen OB, Madsen TS, Falke JJ (1997) Intermolecular tuning of calmodulin by target peptides and proteins: differential effects on Ca2+ binding and implications for kinase activation. Protein Sci 6: 794–807. 10.1002/pro.5560060406 9098889 PMC2144748

[7] Bayley PM, Findlay WA, Martin SR (1996) Target recognition by calmodulin: dissecting the kinetics and affinity of interaction using short peptide sequences. Protein Sci 5: 1215–28. 10.1002/pro.5560050701 8819155 PMC2143466

[8] Gaertner TR, A PJ, Waxham MN (2004) RC3/Neurogranin and Ca2+/calmodulin-dependent protein kinase II produce opposing effects on the affinity of calmodulin for calcium. J Biol Chem 279: 39374–82. 10.1074/jbc.M405352200 15262982

[9] Evans TIA, Shea MA (2009) Energetics of Calmoudlin Domain Interactions with the Calmodulin Binding Domain of CaMKII. Proteins: Struct, Funct, Bioinf 76: 47–61. 10.1002/prot.22317 19089983 PMC2924166

[10] Feldkamp MD, Yu L, Shea MA (2011) Structural and Energetic Determinants of Apo Calmodulin Binding to the IQ Motif of the NaV 1.2 Voltage-Dependent Sodium Channel. Structure 19: 733–747. 10.1016/j.str.2011.02.009 21439835 PMC3094505

[11] Kim SA, Heinze KG, Bacia K, Waxham MN, Schwille P (2005) Two-photon cross-correlation analysis of intracellular reactions with variable stoichiometry. Biophys J 88: 4319–36. 10.1529/biophysj.104.055319 15792970 PMC1305661

[12] Theoharis NT, Sorensen BR, Theisen-Toupal J, Shea MA (2008) The neuronal voltage-dependent sodium channel type II IQ motif lowers the calcium affinity of the C-domain of calmodulin. Biochemistry 47: 112–23. 10.1021/bi7013129 18067319

[13] O’Donnell SE, Yu L, Fowler CA, Shea MA (2011) Recognition of β-calcineurin by the domains of calmodulin: thermodynamic and structural evidence for distinct roles. Proteins: Struct, Funct, Bioinf 79: 765–86. 10.1002/prot.22917 21287611 PMC3057930

[14] Monod JL, Wyman J, Changeux JP (1965) On the Nature of Allosteric Transitions: A Plausible Model. J Mol Biol 12: 88–118. 10.1016/S0022-2836(65)80285-6 14343300

[15] Rubin MM, Changeux JP (1966) On the nature of allosteric transitions: implications of non-exclusive ligand binding. J Mol Biol 21: 265–74. 10.1016/0022-2836(66)90097-0 5972463

[16] Malmendal A, Evenäas J, Forsén S, Akke M (1999) Structural dynamics in the C-terminal domain of calmodulin at low calcium levels. J Mol Biol 293: 883–99. 10.1006/jmbi.1999.3188 10543974

[17] Evenäas J, Forsén S, Malmendal A, Akke M (1999) Backbone dynamics and energetics of a calmodulin domain mutant exchanging between closed and open conformations. J Mol Biol 289: 603–17. 10.1006/jmbi.1999.2770 10356332

[18] Evenäas J, Malmendal A, Akke M (2001) Dynamics of the transition between open and closed conformations in a calmodulin C-terminal domain mutant. Structure 9: 185–95. 10.1016/S0969-2126(01)00575-5 11286885

[19] Chen YG, Hummer G (2007) Slow conformational dynamics and unfolding of the calmodulin C-terminal domain. J Am Chem Soc 129: 2414–5. 10.1021/ja067791a 17290995

[20] Tripathi S, Portman JJ (2009) Inherent flexibility determines the transition mechanisms of the EF-hands of calmodulin. Proc Natl Acad Sci USA 106: 2104–2109. 10.1073/pnas.0806872106 19190183 PMC2650115

[21] Fallon J, Quiocho F (2003) A Closed Compact Structure of Native Ca2+-Calmodulin. Structure 11: 1303–1307. 10.1016/j.str.2003.09.004 14527397

[22] Tan RY, Mabuchi Y, Grabarek Z (1996) Blocking the Ca2+-induced conformational transitions in calmodulin with disulfide bonds. J Biol Chem 271: 7479–83. 10.1074/jbc.271.13.7479 8631777

[23] Meyer DF, Mabuchi Y, Grabarek Z (1996) The Role of Phe-92 in the Ca2+ -induced Conformational Transition in the C-terminal Domain of Calmodulin. Biochemistry 271: 11284–11290. 8626680 10.1074/jbc.271.19.11284

[24] Ababou A, Desjarlais JR (1996) Solvation energetics and conformational change in EF-hand proteins. Protein Sci 2: 301–312. 11266616 10.1110/ps.33601 PMC2373930

[25] Ikura M, Ames JB (2006) Genetic polymorphism and protein conformational plasticity in the calmodulin superfamily: two ways to promote multifunctionality. Proc Natl Acad Sci USA 103: 1159–64. 10.1073/pnas.0508640103 16432210 PMC1360552

[26] Stefan MI, Edelstein SJ, Le Novère N (2008) An allosteric model of calmodulin explains differential activation of PP2B and CaMKII. PNAS 31 10.1073/pnas.0804672105 18669651 PMC2504824

[27] Finn BE, Evenas J, Drakenberg T, Waltho JP, Thulin E, et al (1995) Calcium-induced structural changes and domain authonomy in calmodulin. Nat Struct Biol 2: 777–783. 10.1038/nsb0995-777 7552749

[28] Minowa O, Yagi K (1984) Calcium binding to tryptic fragments of calmodulin. J Biochem 96: 1175–82. 6520119 10.1093/oxfordjournals.jbchem.a134935

[29] Linse S, Helmersson A, Forsén S (1991) Calcium binding to calmodulin and its globular domains. J Biol Chem 266: 8050–4. 1902469

[30] Nelson MR, Thulin E, Fagan PA, Forséen S, Chazin WJ (2002) The EF-hand domain: A globally cooperative structural unit. Protein Sci 11: 198–205. 10.1110/ps.33302 11790829 PMC2373453

[31] VanScyoc WS, Sorensen BR, Rusinova E, Laws WR, Ross JB, et al (2002) Calcium binding to calmodulin mutants monitored by domain-specific intrinsic phenylalanine and tyrosine fluorescence. Biophys J 83: 2767–80. 10.1016/S0006-3495(02)75286-7 12414709 PMC1302361

[32] Faas GC, Raghavachari S, Lisman JE, Mody I (2011) Calmodulin as a direct detector of Ca2+ signals. Nat Neurosci 14: 301–4. 10.1038/nn.2746 21258328 PMC3057387

[33] Drake SK, Falke JJ (1996) Kinetic Tuning of the EF-Hand Calcium Binding Motif: The Gateway Residue Independently Adjusts (i) Barrier Height and (ii) Equilibrium. Biochemistry 35: 1753–1760. 10.1021/bi952335c 8639655

[34] Drake SK, Zimmer MA, Kundrot C, Falke JJ (1997) Molecular tuning of an EF-hand-like calcium binding loop. Contributions of the coordinating side chain at loop position 3. J Gen Physiol 110: 173–84. 10.1085/jgp.110.2.173 9236210 PMC2233790

[35] Wang BO, Martin SR, Newman RA, Hamilton SL, Shea MA, et al (2004) Biochemical properties of V91G calmodulin: A calmodulin point mutation that deregulates muscle contraction in Drosophila. Protein Sci 13: 3285–3297. 10.1110/ps.04928204 15557269 PMC2287309

[36] Kubota Y, Putkey Ja, Waxham MN (2007) Neurogranin controls the spatiotemporal pattern of postsynaptic Ca2+/CaM signaling. Biophys J 93: 3848–59. 10.1529/biophysj.107.106849 17704141 PMC2084249

[37] Cui Y, Wen J, Sze KH, Man D, Lin D, et al (2003) Interaction between calcium-free calmodulin and IQ motif of neurogranin studied by nuclear magnetic resonance spectroscopy. Anal Biochem 315: 175–182. 10.1016/S0003-2697(03)00007-1 12689827

[38] Chichili VPR, Xiao Y, Seetharaman J, Cummins TR, Sivaraman J (2013) Structural Basis for the Modulation of the Neuronal Voltage-Gated Sodium Channel NaV1.6 by Calmodulin. Sci Rep 3 10.1038/srep02435 23942337 PMC3743062

[39] Stefan MI, Edelstein SJ, Le Novère N (2009) Computing phenomenologic Adair-Klotz constants from microscopic MWC parameters. BMC Syst Biol 68 10.1186/1752-0509-3-68 19602261 PMC2732593

[40] Li C, Donizelli M, Rodriguez N, Dharuri H, Endler L, et al (2010) BioModels Database: An enhanced, curated and annotated resource for published quantitative kinetic models. BMC Sys Bio 4: 92 10.1186/1752-0509-4-92 20587024 PMC2909940

[41] Johnson JD, Snyder C, Walsh M, Flynn M (1996) Effects of myosin light chain kinase and peptides on Ca2+ exchange with the N- and C-terminal Ca2+ binding sites of calmodulin. J Biol Chem 271: 761–7. 10.1074/jbc.271.2.817 8557684

[42] Drabikowski W, Brzeska H, Venyaminov SY (1982) Tryptic Fragments of Calmodulin. J Biol Chem 257: 11584–11590. 6811583

[43] Kuboniwa H, Tjandra N, Grzesiek S, Ren H, Klee CB, et al (1995) Solution structure of calcium-free calmodulin. Nat Struct Biol 2: 768–776. 10.1038/nsb0995-768 7552748

[44] Chou JJ, Li S, Klee CB, Bax A (2001) Solution structure of Ca(2+)-calmodulin reveals flexible hand-like properties of its domains. Nat Struct Biol 8: 990–7. 10.1038/nsb1101-990 11685248

[45] Stigler J, Rief M (2012) Calcium-dependent folding of single calmodulin molecules. PNAS 109: 17814–17819. 10.1073/pnas.1201801109 22753517 PMC3497792

[46] Borsi V, Luchinat C, Parigi G (2009) Global and local mobility of apocalmodulin monitored through fast-field cycling relaxometry. Biophys J 97: 1765–71. 10.1016/j.bpj.2009.07.005 19751682 PMC2749786

[47] Schumacher M, Rivard AF, Bachinger HP, Adelman JP (2001) Structure of the gating domain of a Ca2+-activated K+ channel complexed with Ca2+/calmodulin. Nature 410: 1120–1124. 10.1038/35074145 11323678

[48] Martin SR, Bayley PM, Drakenberg T, Forsén S (1985) Kinetics of calcium dissociation from calmodulin and its tryptic fragments. A stopped-flow fluorescence study using Quin 2 reveals a two-domain structure. Eur J Biochem 151: 543–50. 10.1111/j.1432-1033.1985.tb09137.x 4029146

[49] Martin SR, Bayley PM (1986) The effects of ca2+ and cd2+ on the secondary and tertiary structure of bovine testis calmodulin. a circular-dichroism study. Biochem J 238: 485–490. 3800949 10.1042/bj2380485 PMC1147160

[50] Ulmer TS, Soelaiman S, Li S, Klee CB, Tang WJ, et al (2003) Calcium dependence of the interaction between calmodulin and anthrax edema factor. J Biol Chem 278: 29261–6. 10.1074/jbc.M302837200 12724328

[51] Persechini A, Stemmer PM (2002) Calmodulin is a limiting factor in the cell. Trends Cardiovasc Med 12: 32–7. 10.1016/S1050-1738(01)00144-X 11796242

[52] Kumar V, Chichili VPR, Tang X, Sivaraman J (2013) A novel trans conformation of ligand-free calmodulin. PLoS One 8: e54834 10.1371/journal.pone.0054834 23382982 PMC3558517

[53] Putkey JA, Kleerekoper Q, Gaertner TR, Waxham MN (2003) A new role for IQ motif proteins in regulating calmodulin function. J Biol Chem 278: 49667–70. 10.1074/jbc.C300372200 14551202

[54] Ogawa Y, Tanokura M (1984) Calcium Binding to Calmodulin: Effects of Ionic Strength, Mg2+, pH and Temperature. J Biochem 95: 19–28. 6706907 10.1093/oxfordjournals.jbchem.a134584

[55] Martin SR, Masino L, Bayley PM (2000) Enhancement by Mg2+ of domain specificity in Ca2+- dependent interactions of calmodulin with target sequences. Protein Sci 9: 2477–88. 10.1110/ps.9.12.2477 11206069 PMC2144519

[56] Martinez KL, Corringer PJ, Edelstein SJ, Changeux JP, Mérola F (2000) Structural differences in the two agonist binding sites of the Torpedo nicotinic acetylcholine receptor revealed by time-resolved fluorescence spectroscopy. Biochemistry 39: 6979–90. 10.1021/bi992811p 10841780

[57] Runarsson TP (2000) Stochastic ranking for constrained evolutionary optimization. IEEE Trans Evol Comput 4: 284–294. 10.1109/4235.873238

[58] Edelstein SJ, Stefan MI, Le Novère N (2010) Ligand depletion in vivo modulates the dynamic range and cooperativity of signal transduction. PLoS One 5: e8449 10.1371/journal.pone.0008449 20052284 PMC2797075

[59] Kumar V, Chichili VPR, Zhong L, Tang X, Velazquez-Campoy A, et al (2013) Structural basis for the interaction of unstructured neuron specific substrates neuromodulin and neurogranin with Calmodulin. Sci Rep 3: 1392 10.1038/srep01392 23462742 PMC3589724

[60] Ikura M, Clore GM, Gronenborn AM, Zhu G, Klee CB, et al (1992) Solution structure of a calmodulin-target peptide complex by multidimensional NMR. Science 256: 632–8. 10.1126/science.1585175 1585175

[61] Van der Spoel D, De Groot BL, Hayward S, Berendsen HJC, Vogel H (1996) Bending of the Calmodulin central helix: A theoretical study. Protein Sci 5: 2044–2053. 10.1002/pro.5560051011 8897605 PMC2143272

[62] Jiang J, Zhou Y, Zou J, Chen Y, Patel P, et al (2010) Site-specific modification of calmodulin Ca2+ affinity tunes the skeletal muscle ryanodine receptor activation profile. Biochem J 432: 89–99. 10.1042/BJ20100505 20815817

[63] Chen Y, Zhou Y, Lin X, Wong HC, Xu Q, et al (2011) Molecular interaction and functional regulation of connexin50 gap junctions by calmodulin. Biochem J 435: 711–22. 10.1042/BJ20101726 21320072 PMC3351833

[64] Zhou Y, Tzeng WP, Wong HC, Ye Y, Jiang J, et al (2010) Calcium-dependent association of calmodulin with the rubella virus nonstructural protease domain. J Biol Chem 285: 8855–68. 10.1074/jbc.M109.097063 20086014 PMC2838307

[65] Porumb T, Yau P, Harvey TS, Ikura M (1994) A calmodulin-target peptide hybrid molecule with unique calcium-binding properties. Protein Eng 7: 109–15. 10.1093/protein/7.1.109 8140087

