# Supplementary figures and images for: Modulation of Calmodulin Lobes by Different Targets: An Allosteric Model with Hemiconcerted Conformational Transitions
Source: PLoS Comput Biol. 2015 Jan 22;11(1):e1004063. doi: 10.1371/journal.pcbi.1004063 (PMC4303274; doi:10.1371/journal.pcbi.1004063)

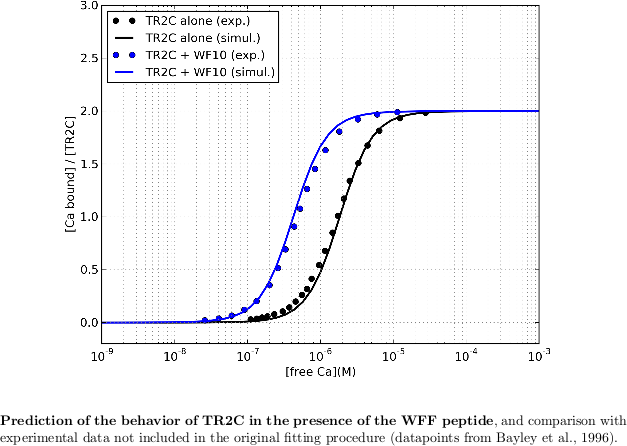

Supplement: S1 Fig — (TIF) [file pcbi.1004063.s001.tif]

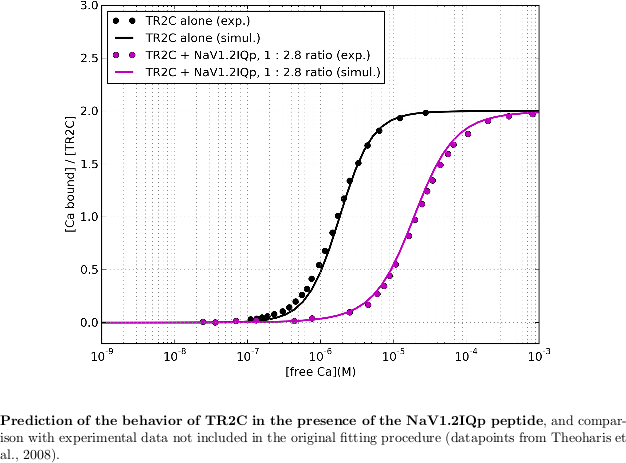

Supplement: S2 Fig — (TIF) [file pcbi.1004063.s002.tif]
