# Supplementary material for: Modulation of Calmodulin Lobes by Different Targets: An Allosteric Model with Hemiconcerted Conformational Transitions
Source: PLoS Comput Biol. 2015 Jan 22;11(1):e1004063. doi: 10.1371/journal.pcbi.1004063 (PMC4303274; doi:10.1371/journal.pcbi.1004063)
Supplement: S1 Table — (PDF) [file pcbi.1004063.s003.pdf]

**Summary of the experimental conditions under which calcium titrations were performed.**

| Reference | Experimental conditions |
|---|---|
| [6] | 20 mM MOPS, 100 mM KCl, 0.5 mM MgCl2, pH 7.4, at 20 – 25°C |
| [7] | 25 mM Tris, 100 mM KCl, pH 7.5 at 30C |
| [9] | 50 mM HEPES, 100 mM KCl, 1 mM MgCl2, 0.05 mM EGTA, 5 mM NTA, pH 7.4 |
| [12] | 50 mM HEPES, 100 mM KCl, 5 mM NTA, 0.05 mM EGTA, 1 mM MgCl2, pH 7.4 at 22°C |
| [22] | 100 mM HEPES, 100 mM KCl, 1 or 2 mM EGTA, 1 mM NTA, pH 7.5 |
| [31] | 50 mM HEPES, 100 mM KCl, 0.05 mM EGTA, 5 mM NTA, pH 7.40, at 22°C |
| [62] | 50 mM HEPES, 100 mM KCl, 5 mM NTA and 0.05 mM EGTA pH 7.4, at 22°C |
| [65] | 50 mM HEPES, 100 mM KCl, pH 7.5, at 25°C |

Importantly, there is a good level of uniformity in the conditions of temperature, pH, and ionic strength. Where temperature was not reported, it is reasonable to assume room temperature, in the range 20 – 25°C
