# Supplementary material for: Modulation of Calmodulin Lobes by Different Targets: An Allosteric Model with Hemiconcerted Conformational Transitions
Source: PLoS Comput Biol. 2015 Jan 22;11(1):e1004063. doi: 10.1371/journal.pcbi.1004063 (PMC4303274; doi:10.1371/journal.pcbi.1004063)
Supplement: S2 Table — (PDF) [file pcbi.1004063.s004.pdf]

Summary of the parameters for the computational model of TR2C.

Parameter	Description	Value	Source
L	Allosteric constant	8616.61	This paper
c	ratio of affinities	0.000317019	This paper
lC	Allosteric constant when C site is occupied	2.73163	Calculated as lC = L*c
lD	Allosteric constant when D site is occupied	2.73163	Calculated as lC = L*c
lCD	Allosteric constant when C and D site are occupied	0.000865977	Calculated as lC = L*c*c
KCR	calcium affinity of site C in the R state	$1.97874e-08$	This paper
KDR	calcium affinity of site D in the R state	$1.97874e-08$	This paper
KCT	calcium affinity of site C in the T state	$6.24171e-05$	This paper
KDT	calcium affinity of site C in the T state	$6.24171e-05$	This paper
KdpepR	peptide affinity for the R state	$7.6e-08$	Bayley et al., 1996
KdpepT	peptide affinity for the R state	$1.1e-05$	Bayley et al., 1996
