# Supplementary material for: Modulation of Calmodulin Lobes by Different Targets: An Allosteric Model with Hemiconcerted Conformational Transitions
Source: PLoS Comput Biol. 2015 Jan 22;11(1):e1004063. doi: 10.1371/journal.pcbi.1004063 (PMC4303274; doi:10.1371/journal.pcbi.1004063)
Supplement: S3 Table — (PDF) [file pcbi.1004063.s005.pdf]

Summary of the chemical species in the TR2C model. The model for the N-lobe is formally analogous to that of TR2C.

Species name	Description
ca	calcium ion
pep	generic name for target peptides (e.g. WFF, WF10, Nav1.2IQp)
R_0	calmodulin in the R state with no calcium bound
R_C	calmodulin in the R state with calcium bound to site C
R_D	calmodulin in the R state with calcium bound to site D
R_CD	calmodulin in the R state with calcium bound to site C and D
T_0	calmodulin in the T state with no calcium bound
T_C	calmodulin in the T state with calcium bound to site C
T_D	calmodulin in the T state with calcium bound to site D
T_CD	calmodulin in the T state with calcium bound to site C and D
pep_R_0	peptide-bound calmodulin in the R state with no calcium bound
pep_R_C	peptide-bound calmodulin in the R state with calcium bound to site C
pep_R_D	peptide-bound calmodulin in the R state with calcium bound to site D
pep_R_CD	peptide-bound calmodulin in the R state with calcium bound to site C and D
pep_T_0	peptide-bound calmodulin in the T state with no calcium bound
pep_T_C	peptide-bound calmodulin in the T state with calcium bound to site C
pep_T_D	peptide-bound calmodulin in the T state with calcium bound to site D
pep_T_CD	peptide-bound calmodulin in the T state with calcium bound to sites C and D
