# Supplementary material for: Modulation of Calmodulin Lobes by Different Targets: An Allosteric Model with Hemiconcerted Conformational Transitions
Source: PLoS Comput Biol. 2015 Jan 22;11(1):e1004063. doi: 10.1371/journal.pcbi.1004063 (PMC4303274; doi:10.1371/journal.pcbi.1004063)
Supplement: S4 Table — (PDF) [file pcbi.1004063.s006.pdf]

**Summary of the reactions used in the TR2C model.**

| Reaction name | Description |
|---|---|
| pep binding to R_0 | pep + R_0 $\rightleftarrows$ pep_R_0 |
| pep binding to R_C | pep + R_C $\rightleftarrows$ pep_R_C |
| pep binding to R_D | pep + R_D $\rightleftarrows$ pep_R_D |
| pep binding to R_CD | pep + R_CD $\rightleftarrows$ pep_R_CD |
| pep binding to T_0 | pep + T_0 $\rightleftarrows$ pep_T_0 |
| pep binding to T_C | pep + T_C $\rightleftarrows$ pep_T_C |
| pep binding to T_D | pep + T_D $\rightleftarrows$ pep_T_D |
| pep binding to T_CD | pep + T_CD $\rightleftarrows$ pep_T_CD |
| ca binding to R_0 on site C | ca + R_0 $\rightleftarrows$ R_C |
| ca binding to R_0 on site D | ca + R_0 $\rightleftarrows$ R_D |
| ca binding to R_C on site D | ca + R_C $\rightleftarrows$ R_CD |
| ca binding to R_D on site C | ca + R_D $\rightleftarrows$ R_CD |
| ca binding to T_0 on site C | ca + T_0 $\rightleftarrows$ T_C |
| ca binding to T_0 on site D | ca + T_0 $\rightleftarrows$ T_D |
| ca binding to T_C on site D | ca + T_C $\rightleftarrows$ T_CD |
| ca binding to T_D on site C | ca + T_D $\rightleftarrows$ T_CD |
| ca binding to pep_R_0 on site C | ca + pep_R_0 $\rightleftarrows$ pep_R_C |
| ca binding to pep_R_0 on site D | ca + pep_R_0 $\rightleftarrows$ pep_R_D |
| ca binding to pep_R_C on site D | ca + pep_R_C $\rightleftarrows$ pep_R_CD |
| ca binding to pep_R_D on site C | ca + pep_R_D $\rightleftarrows$ pep_R_CD |
| ca binding to pep_T_0 on site C | ca + pep_T_0 $\rightleftarrows$ pep_T_C |
| ca binding to pep_T_0 on site D | ca + pep_T_0 $\rightleftarrows$ pep_T_D |
| ca binding to pep_T_C on site D | ca + pep_T_C $\rightleftarrows$ pep_T_CD |
| ca binding to pep_T_D on site C | ca + pep_T_D $\rightleftarrows$ pep_T_CD |
| Conformational transition T_0 / R_0 | T_0 $\rightleftarrows$ R_0 |
| Conformational transition T_C / R_C | T_0 $\rightleftarrows$ R_0 |
| Conformational transition T_D / R_D | T_0 $\rightleftarrows$ R_0 |
| Conformational transition T_CD / R_CD | T_0 $\rightleftarrows$ R_0 |
| Conformational transition pep_T_0 / pep_R_0 | pep_T_0 $\rightleftarrows$ pep_R_0 |
| Conformational transition pep_T_C / pep_R_C | pep_T_C $\rightleftarrows$ pep_R_C |
| Conformational transition pep_T_D / pep_R_D | pep_T_D $\rightleftarrows$ pep_ R_D |
| Conformational transition pep_T_CD / pep_R_CD | pep_T_CD $\rightleftarrows$ pep_ R_CD |

All listed reactions are reversible. The model for the N-lobe is formally analogous to that of TR2C.
