# Supplementary material for: Modulation of Calmodulin Lobes by Different Targets: An Allosteric Model with Hemiconcerted Conformational Transitions
Source: PLoS Comput Biol. 2015 Jan 22;11(1):e1004063. doi: 10.1371/journal.pcbi.1004063 (PMC4303274; doi:10.1371/journal.pcbi.1004063)
Supplement: S5 Table — (PDF) [file pcbi.1004063.s007.pdf]

Summary of the independent parameters for the model of intact calmodulin.

Parameter	Description	Value	Source
lC	Allosteric constant for the C-lobe	8616.61	this paper
lN	Allosteric constant for the N-lobe	398000	this paper
cC	defined as KCR / KCT	0.000159	this paper
cN	defined as KAR / KAT	0.000215	this paper
KAR	Ca affinity of site A in the R state	1.97628e-08	Calculated as KAT*cN
KAT	Ca affinity of site A in the T state	9.192e-05	this paper
KBR	Ca affinity of site B in the R state	1.97628e-08	Calculated as KBT*cN
KBT	Ca affinity of site B in the T state	9.192e-05	this paper
KCR	Ca affinity of site C in the R state	1.98496e-08	Calculated as KCT*cC
KCT	Ca affinity of site C in the T state	0.00012484	this paper
KDR	Ca affinity of site D in the R state	1.98496e-08	Calculated as KDT*cC
KDT	Ca affinity of site C in the T state	0.00012484	this paper
eCR_tbp	Kd_tbp_RR / Kd_tbp_RT	(target-dependent)	/
eCT_tbp	Kd_tbp_TR / Kd_tbp_TT	(target-dependent)	/
eNR_tbp	Kd_tbp_RR / Kd_tbp_TR	(target-dependent)	/
eNT_tbp	Kd_tbp_RT / Kd_tbp_TT	(target-dependent)	/
eCR_rbp	Kd_tbp_RR / Kd_tbp_RT	(target-dependent)	/
eCT_rbp	Kd_tbp_TR / Kd_tbp_TT	(target-dependent)	/
eNR_rbp	Kd_tbp_RR / Kd_tbp_TR	(target-dependent)	/
eNT_rbp	Kd_tbp_RT / Kd_tbp_TT	(target-dependent)	/

The dummy names "tbp" and "rbp" indicate the generic targets that bind preferably to the T and R states, respectively.The full model of intact calmodulin contains over 500 reversible reactions (or more if additional targets are included), therefore they are not listed here for brevity. The SBML model can however be retrieved from Biomodels Database (model ID: MODEL1405060000)
